# Virtual Reality during Intrathecal Pump Refills in Children: A Case Series

**DOI:** 10.3390/jcm11195877

**Published:** 2022-10-05

**Authors:** Lisa Goudman, Julie Jansen, Ann De Smedt, Maxime Billot, Manuel Roulaud, Philippe Rigoard, Maarten Moens

**Affiliations:** 1 STIMULUS Research Group, Vrije Universiteit Brussel, Laarbeeklaan 103, 1090 Brussels, Belgium; 2 Department of Neurosurgery, Universitair Ziekenhuis Brussel, Laarbeeklaan 101, 1090 Brussels, Belgium; 3 Center for Neurosciences (C4N), Vrije Universiteit Brussel, Laarbeeklaan 103, 1090 Brussels, Belgium; 4 Pain in Motion (PAIN) Research Group, Department of Physiotherapy, Human Physiology and Anatomy, Faculty of Physical Education and Physiotherapy, Vrije Universiteit Brussel, Laarbeeklaan 103, 1090 Brussels, Belgium; 5 Research Foundation—Flanders (FWO), 1090 Brussels, Belgium; 6 Department of Physical Medicine and Rehabilitation, Universitair Ziekenhuis Brussel, Laarbeeklaan 101, 1090 Brussels, Belgium; 7 PRISMATICS Lab (Predictive Research in Spine/Neuromodulation Management and Thoracic Innovation/Cardiac Surgery), Poitiers University Hospital, 86021 Poitiers, France; 8 Department of Spine Surgery & Neuromodulation, Poitiers University Hospital, 86021 Poitiers, France; 9 Pprime Institute UPR 3346, CNRS, ISAE-ENSMA, University of Poitiers, 86360 Poitiers, France; 10 Department of Radiology, Universitair Ziekenhuis Brussel, Laarbeeklaan 101, 1090 Brussels, Belgium

**Keywords:** immersive technology, distraction, neuromodulation, personalized care, children

## Abstract

Virtual reality has proven to be an effective approach to decrease pain in acute settings, both in adults and children. The aim of this study is to evaluate whether virtual reality (VR) could reduce pain during an intrathecal pump refill procedure in children receiving intrathecal drug delivery, compared to a standard refill procedure. This is a three-arm crossover randomized controlled trial, evaluating the effect of VR on pain in children with cerebral palsy undergoing an intrathecal pump refill compared to a standard refill and a refill with distraction (watching a video). Pain was evaluated using the Wong–Baker Faces Scale. Secondary outcomes were procedural pain, fear, state anxiety, the incidence of adverse events and satisfaction. Six children participated in this study, whereby all children underwent the three conditions. Five children indicated an equal of lower pain score during VR, compared to a standard refill. This finding of an equal or lower pain intensity score for the VR condition compared to the control condition was also revealed by the ratings of the parents, physician and the researcher. The influence of VR on anxiety and fear seem to be in line with the influence of watching a video. In terms of satisfaction, all children and parents agreed with the statement that they would like to use VR again for a next refill. Due to the lack of adverse events, the high degree of satisfaction of children with VR and the decreased pain levels after a refill with VR, physicians may aim to explore the implementation of VR during intrathecal pump refill procedures in children in a daily clinical routine care setting.

## 1. Introduction

Most children experience pain and fear when receiving a medical treatment; two feelings which are closely related and affecting one another [1]. Specifically for children requiring care with repeated hospital visits, experiences of fear and pain are often reported [2], pointing out that fear plays an important role in the pain experience related to needle procedures [3,4,5]. When children are facing surgery, almost 50% of the children suffer from anxiety [6,7], whereby nonpharmacological modalities such as video games or cartoons can reduce preoperative anxiety [8,9,10].

There is mounting evidence from both acute pain conditions, such as wound care, and chronic pain settings, that virtual reality (VR) could play a role as an additional treatment method to relieve pain or reduce anxiety [11,12,13,14]. VR is a technological rehabilitation tool that allows the user to experience interaction with a computer-generated environment [15], thereby leading to a high degree of immersion and real-time interaction [16]. It constitutes an enriched environment with augmented multiple sensory feedbacks (auditory, visual, tactile VR enriched environment) that has already demonstrated beneficial effects during pediatric procedures among which intravenous line placement [4], preoperative preparation of children undergoing general anesthesia [16] and during burn wound care [17]. A possible explanation for its mechanism of action is provided by “the gate-theory of attention” [18]. VR reduces the perception of pain by diverting attention away from the pain [19].

Administration of intrathecal baclofen (ITB) through an implantable drug delivery system provides a suitable therapy option to decrease intractable severe spasticity [20]. This involves the implantation of an intrathecal catheter which is connected to a subcutaneous pump [20]. Children who have been implanted with an intrathecal (IT) pump need to come to the hospital for regular evaluation and a refill approximately every 3–6 months, depending on the exact dose [21]. Successful ITB pump therapy thus requires continuous input from specialist services to maintain an effective and safe therapy. During the refill, the physician places a needle directly into the reservoir to refill the pump (i.e., refill through a transcutaneous needle) [21]. To alleviate the pain and fear with these refill procedures, it is hypothesized that VR could alleviate pain and make these regular refills more feasible. Therefore, the aim of this study is to evaluate whether VR reduces pain during an IT pump refill procedure in children receiving intrathecal drug delivery, compared to a standard refill procedure.

## 2. Materials and Methods

### 2.1. Participants

In this study, children with cerebral palsy between 8 and 16 years (male and female) who received intrathecal baclofen delivery through an implanted pump (SynchroMed II, Medtronic Inc., Minneapolis, MN, USA) were recruited from Universitair Ziekenhuis Brussel by the treating physician. Children were only eligible for inclusion if the child and parent were able to speak Dutch or French for the questionnaires. Additionally, cognitive and language functioning enabling communication between the physician/researcher and the child was an inclusion criterium. Exclusion criteria were: (1) children with susceptibility to motion sickness or cyber-sickness, (2) children with susceptibility to claustrophobia and (3) children with a history of seizures/epilepsy. Both the children and parents were asked if the child would be willing to participate in the study. Two different informed consents were created, one for parents and one for the child, which was written in a children-adapted way. As such, both the child and the parents received an informed consent and both provided written informed consent before participating in the study.

Prior to the study, approval for the conduct of the trial was obtained from the Ethics Committee of the University Hospital (BUN 1432020000308) on 6 January 2021. The study was registered on clinicaltrials.gov (NCT04737668).

### 2.2. Protocol

This was a prospective experimental single-center three-arm crossover randomized controlled trial, investigating the effect of VR on pain in children undergoing an intrathecal pump refill compared to standard care and distraction. The study was conducted during three subsequent normal hospital visits for regular pump refill at the hospital. Children and parents who agreed to participate and who provided written informed consent to participate received three different pump refills (three conditions) in a randomized order. The first condition consisted of a pump refill as usual, the second condition consisted of a refill with distraction through a video and the third condition through a refill with a VR game. During a normal refill, i.e., standard care, the pump refill was performed as under normal circumstances (no form of sedation or topical anaesthesia). During the distraction condition, children were able to watch a commercial video on YouTube [22], according to their own preference. During the VR condition, children played a commercially available VR game for children, called *“All-Star Fruit Racing”*. In this game, children raced through fruit-filled tracks while trying to smash fruit, drift round corners and tear up the tracks. The pain control virtual environment was delivered through the PICO VR goggle. The technological equipment included a VR goggle with a motion tracker. While moving their heads, the children moved the racing car and played the game.

The conditions were randomised through a computer-generated randomization list. The list with patient numbers and the randomization order that resulted from this randomization procedure were stored in a sealed envelope.

### 2.3. Outcome Measurements

**Figure 1:**
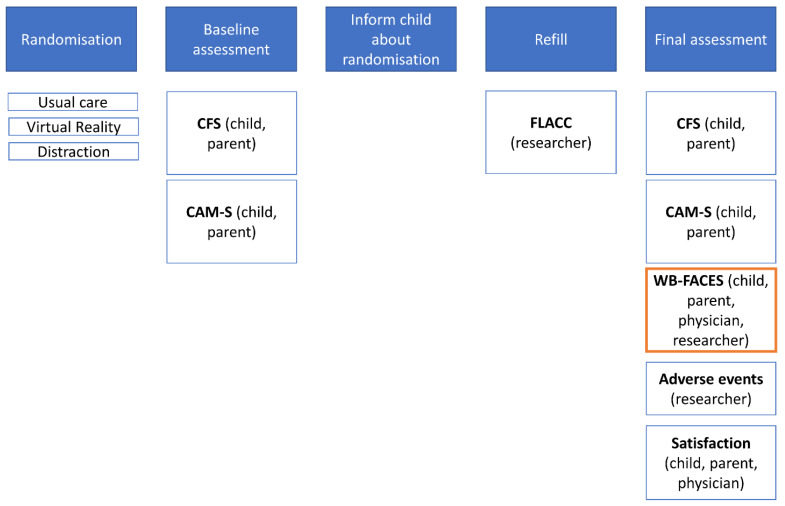
Schematic overview of the study outcome measurements. The primary outcome measure is indicated in orange.

During the first visit, demographics of the child and the parent were collected. Immediately before the refill procedures, fear and anxiety were measured (baseline assessment). Immediately after the refills, fear, anxiety, pain, adverse events and satisfaction were evaluated (Figure 1). During the refill procedure, procedural pain was evaluated.

The primary outcome measurement consisted of an evaluation of the pain through the Wong–Baker Faces (WB-FACES) Scale. Pain scores were assessed using self-reports (child), parent’s reports, the reports of the physician who performed the refill procedure and the external researcher. The WB-FACES is consistent with the numeric rating scale of 0 to 10. Six faces on the scale show a range of emotions from smiling (0 = very happy/no pain) to crying (10 = hurts worst). This scale was previously used as a primary outcome measure to evaluate the effect of VR in children [23].

Procedural pain during refill was also assessed using the Face, Legs, Activity, Cry, Consolability scale (FLACC), a behavioural observational pain assessment tool shown to be reliable for evaluating procedural pain in children [24]. The scale scores behaviour from 0 to 2 in five categories—face, leg, activity, cry, and consolability (maximum total FLACC score = 10) [20]. FLACC behaviour rating was performed by the external researcher and previously applied in the context of VR in children [22].

The degree of fear experienced by the children was measured using the Child Fear Scale (CFS). This one-item scale consists of five sex-neutral faces, showing fear ratings of 0–4, which were no fear (0), a little fear (1), some fear (2), very fear (3) and extreme fear (4). The children and parents were asked to select the faces that best described the fear levels of the children receiving intravenous injections before and after the refills. The CFS is based on the Faces Anxiety Scale for adults [25]. Assessment of construct validity revealed high concurrent convergent validity with another self-report measure of fear and moderate discriminant validity with child coping behaviour and child distress behaviour [26].

The Children’s Anxiety Meter (CAM) assesses children’s anxiety in clinical settings and before medical procedures. The children and parents completed the CAM before and after the refill procedures. The CAM is drawn like a thermometer with a bulb at the bottom, also including horizontal lines at intervals going up to the top. Each child was asked to mark how he/she felt “right now” to measure state anxiety (CAM-S) [27]. Scores ranged from 0 to 10. An evaluation of the construct validity indicated that children’s CAM-S scores were significantly associated with all parent measures and observed distress ratings, supporting the use of the CAM-S for assessment of child anxiety in clinical settings [28].

The incidence of adverse events was evaluated after the refill until discharge from the hospital by the researcher. The following adverse events were recorded: nausea, vomiting, motion sickness, dizziness, and seizure [29].

Satisfaction was evaluated with two questions, namely “If I were to have the procedure again, I would like to use this VR/video” and “How interested were you in the VR/video”. This question was also provided to the parent, in which “I” is replaced by “my child” and the physician in which “I” is replaced by “my patient” [29]. The questions utilized a 5-point Likert agreement scale with a score of 1 meaning “completely disagree” and 5 meaning “completely agree.” This question was asked both after the distraction and VR condition.

### 2.4. Statistical Analysis

Descriptive statistics were provided as mean (±standard deviation), median (Q1–Q3) or as an absolute number of observations (percentage). Due to the limited number of participants, no statistical inferences were made.

## 3. Results

### 3.1. Descriptive Statistics

In total, six children were included in this study, whereby all children underwent the three refill conditions (no missing data). The first study participant was recruited on 10 February 2021 Afterwards, participants were consecutively recruited, whereby the last patients was included in the study on 14 July 2021. All refill procedures were conducted between 10 February 2021 and 26 January 2022. Four girls and two boys took part in the study, with a mean age of 13.2 years. Individual patient demographics are presented in Table 1.

### 3.2. Primary Outcome Measurement: Pain

**Figure 2:**
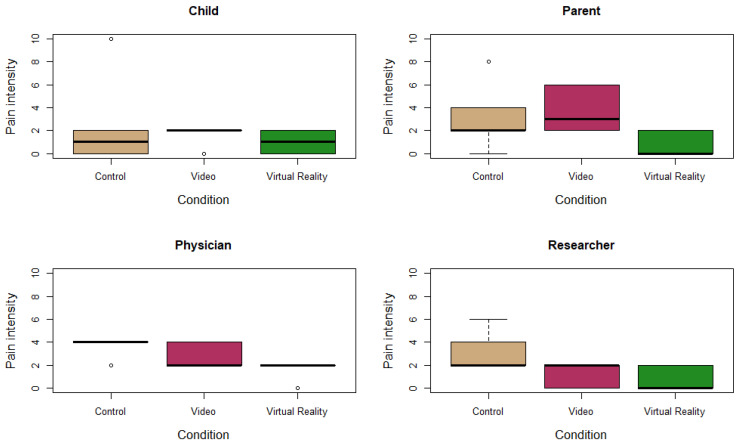
Pain ratings using the Wong–Baker Faces (WB-FACES) Scale for children, parents, physician and researcher after the three refill conditions: standard refill (brown), refill with video (pink) and refill with virtual reality (green).

Two of the children indicated a similar pain intensity score for all three conditions. One child indicated a score of 0 for the control condition and a score of 2 for the video and VR condition, while another child mentioned a score of 0 for the control and VR condition and a score of 2 for the video condition. The remaining two children indicated a lower score for the VR condition compared to the control condition. Parents indicated a similar or lower score for the VR condition compared to the control condition, a finding that was also revealed by the pain intensity ratings of the physician and the researcher. Table 1 presents an overview of all pain intensity ratings per child. In terms of median values, the median pain intensity score for the control condition versus the VR condition was 1 (Q1–Q3: 0–2) versus 1 (Q1–Q3: 0–2) for children, 2 (Q1–Q3: 2–3.5) versus 0 (Q1–Q3: 0–1.5) for parents, 4 (Q1–Q3: 4–4) versus 2 (Q1–Q3: 2–2) for the physician and 2 (Q1–Q3: 2–3.5) versus 0 (Q1–Q3: 0–1.5) for the researcher. For the refill procedure with distraction, median values of 2 (Q1–Q3: 2–2) were reported by the children, 3 (Q1–Q3: 2–5.5) by parents, 2 (Q1–Q3: 2–3.5) by the physician and 2 (Q1–Q3: 0.5–2) by the researcher. Figure 2 presents an overview of the pain intensity rating ratings per condition.

### 3.3. Secondary Outcome Measurements

Procedural pain was observed by the researcher during the refill procedure, whereby median scores of 2 (Q1–Q3: 1.25–2.75) were revealed for the control condition, 1 (Q1–Q3: 1–1.75) for the video condition and 1 (Q1–Q3: 0.25–1) for the VR condition.

**Figure 3:**
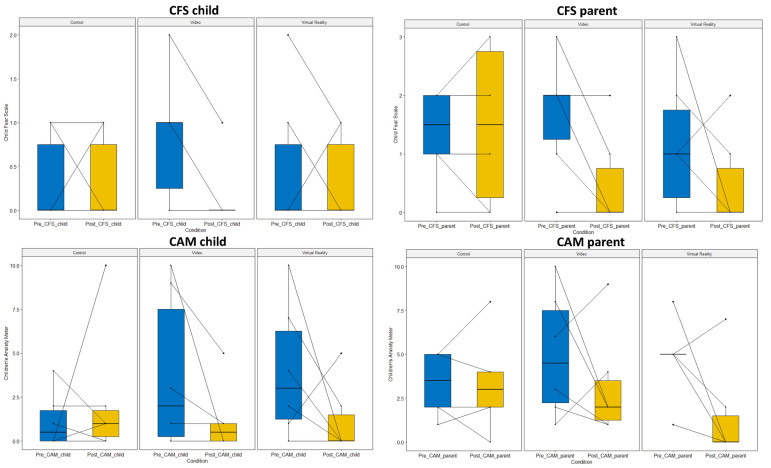
Fear and state anxiety for children and parents after the three refill conditions: standard refill (**left panel**), refill with video (**middle panel**) and refill with virtual reality (**right panel**). The blue boxes represent fear and state anxiety before the refill, while the yellow boxes indicate the status after the refill procedure.

In terms of fear, all children (except one) experienced an equal or lower amount of fear after the procedure compared to before the procedure (Figure 3). For all conditions, total CFS scores ranged between no fear and some fear before the procedure and between no fear and a little fear after the procedure. For parents, scores before and after the refill ranged between no fear and very fear. As observed with the CFS scores of the children, only one parent had an increase in CFS score during the control condition and VR condition (same dyad). The other parents revealed an equal score or lower score after the refill. Based on the visual inspection of the parent CFS reportings after the refill, there seemed to be lower degrees of fear during the video and VR condition.

Regarding state anxiety, scores before the refill ranged from 0 to 10, as were scores after the refill. As observed with CFS, scores before the refill revealed a high variability between the conditions (Figure 3). For two children, state anxiety increased after the refill during the control condition, as was the case for one child in the VR condition. For parents, state anxiety ranged from 1 to 10 before the refill and from 0 to 9 after the refill. Scores increased for two parents in the control and video condition and for one parent in the VR condition. Based on visual inspection of the plots, for parents the VR condition seemed to result in lower state anxiety, while for children the video condition also seemed to be a suitable option to reduce state anxiety.

In terms of satisfaction with the VR or video refill conditions, all children and parents agreed with the statement that they would like to use VR again for a next refill (Table 2).

For the video, one child disagreed with this statement. Based on the ratings of how interested the child was in the VR, both children, parents and the physician agreed that they were interested in the device. For the video, children and parents indicated an interest, while the physician once indicated no interest of the child for the video.

The researcher could not observe any of the following adverse events: nausea, vomiting, motion sickness, dizziness, or seizure.

## 4. Discussion

In this study, we explored the value of VR to reduce pain in children with cerebral palsy who need regular refill procedures for their intrathecal pump. Five children indicated an equal or lower pain score during a refill with VR compared to a standard refill. This finding of an equal or lower pain intensity score for the VR condition compared to the control condition was also revealed by the ratings of the parents, the physician and the researcher.

A recent review about the state of the art of VR indicated that 39.8% of the published articles incorporated VR in the context of acute pain, 34.3% in chronic pain settings and 25.9% in experimental settings [19]. Of the studies related to acute pain, 23.6% were situated in the field of needle insertions, with a primary focus on implementing immersive and interactive VR platforms within acute pain applications [19]. Immersion (i.e., subjective user assessment of being absorbed in a virtual world and the VR configuration [30]), interactivity (i.e., the degree to which a user is capable of influencing the virtual world [30]) and presence (i.e., subjective experience of being in an environment, even when a person is physically situated in another environment [31]) are considered the main pillars of VR applications [32]. The three-dimensional 360-degree virtual environment that was presented through a head-mounted display, combined with an active contribution of the children to the race, has led to an immersive, interactive VR experience in the current study that enabled children to reduce pain, as reported in the majority of studies in acute settings [33,34,35].

The main working mechanism underpinning VR in acute settings is attributed to distraction [13]. Distraction is defined by the engagement of cognitive and attentional resources by the stimuli delivered by VR, with hypoalgesic effects as a result of active competition for resources that are necessary for pain processing [36]. As an active comparator, a distraction condition under the form of a video was added to this experimental study. Both applications presumably work through a similar mechanism, however, the immersion, interactivity and presence are key elements of VR compared to a video. These three components seem to be responsible for the differences in pain intensity scores between the refill during distraction and VR, whereby mainly the ratings of children and parents were different during both conditions. Therefore, it seems that passive distraction by watching a video may be less effective in reducing acute pain, a finding that was also found in burn wound care in adolescents [17].

Since these children are confronted with regular hospital visits, it is expected that they experience fear or anxiety related to the refill procedures. Before the refill, degrees of fear varied from no fear up to some fear and anxiety ranged from no anxiety up to a maximum score of 10 regarding state anxiety, clearly showing the uncomfortable feeling of some children during the hospital visit. It appears that a video as well as a VR application are suitable to reduce fear and anxiety levels or hold them at the same level as before the refill procedure, as scored by both children and parents. Nevertheless, for one child, fear and anxiety increased after the VR condition. This suggests that for psychological outcome variables, a 2D setting seems to be a strong distractor as well, wherefore a 3D setting is not necessary. This result could lead towards the implementation of personalized care for children who need regular hospital visits, in which they can freely choose their preferred choice of distraction in relation to acute medical needle procedures. A previous study on the effect of self-distractors in children and adolescents with cancer in relation to port access or venipuncture indicated that 72% of the children preferred a Nintendo© game, 18% VR glasses, 5% a music table, and 5% preferred that their parents blew bubbles into the room [37], clearly pointing out that non-immersive distractors are not necessarily inferior compared to immersive distractors [38]. In this study, however, children were more inclined to choose a refill with VR than a refill with video, a result that is in contrast to the previous study in which VR glasses received a lower rating. One possible explanation for this result is that children are attracted towards distraction conditions with a high degree of interactivity, such as playing on a Nintendo, compared to watching a music video. For physicians, the addition of VR did not hamper the refill procedure, whereby the physician indicated that children were interested in the VR and that a next refill could be conducted with VR as distraction tool.

The main limitation of this study is the limited number of patients that fulfilled the inclusion and exclusion criteria to participate in this monocentric study. Nevertheless, based on these promising results on satisfaction, pain relief and the lack of adverse events, this study could inspire pain physicians to make small adaptations in their daily clinical care routine with pump refills in children. Larger studies to evaluate the efficacy of this approach should still be performed. Another limitation is that this study was conducted during the ongoing COVID-19 pandemic, whereby most of the children wore a face mask during the hospital visit and the refill procedure. Therefore, the evaluation of the FLACC to assess procedural pain was complicated and should be interpreted with caution.

## 5. Conclusions

Children preferred to have their next intrathecal pump refill with VR goggles. Based on this case series, no adverse events were observed/reported, an equal or lower pain score was revealed, and for most of the children, lower degrees of anxiety and fear were observed with a refill with VR. Therefore, it may be suggested that VR could be used to facilitate the refill procedure in children who are regularly undergoing an intrathecal pump refill in the hospital.

## Figures and Tables

**Table 1:** Individual pain intensity ratings using the Wong–Baker Faces Scale after a normal refill condition, refill with video and refill with virtual reality for children, parents, physician and researcher.

Patient ID	Sex	Age	Condition	Pain Score Child	Pain Score Parent	Pain Score Physician	Pain Score Researcher
1	Male	14	Control	0	2	4	4
			Video	2	4	4	2
			Virtual Reality	2	0	2	2
2	Female	14	Control	2	4	4	2
			Video	2	6	2	0
			Virtual Reality	2	0	0	0
3	Female	16	Control	2	2	2	2
			Video	2	2	2	2
			Virtual Reality	0	0	2	0
4	Male	10	Control	0	2	4	2
			Video	0	2	2	0
			Virtual Reality	0	2	2	0
5	Female	10	Control	10	8	4	6
			Video	2	6	4	2
			Virtual Reality	2	2	2	2
6	Female	15	Control	0	0	4	2
			Video	2	2	2	2
			Virtual Reality	0	0	2	0

**Table 2:** Satisfaction with the refill procedures with video and virtual reality, according to children, parents and the physician.

Topic	Condition	Rating	Child	Parent	Physician
Next refill with video/VR	Video	Completely disagree	1		
		Disagree			
		Neutral	1	1	
		Agree	2	2	4
		Completely agree	2	3	2
	Virtual reality	Completely disagree			
		Disagree			
		Neutral			1
		Agree	2	1	5
		Completely agree	4	5	
Interest of the child in the video/VR	Video	Completely disagree			
		Disagree			
		Neutral			1
		Agree	2	4	4
		Completely agree	4	2	1
	Virtual reality	Completely disagree			
		Disagree			
		Neutral			
		Agree	2	1	6
		Completely agree	4	5	

## Data Availability

The data presented in this study are available on motivated request from the corresponding author.
